# Incorporating virtual reality in undergraduate internal medicine education: a scoping review of current evidence and implementation strategies

**DOI:** 10.1186/s12909-025-08536-2

**Published:** 2026-02-10

**Authors:** Hanaa Mahmoud Nagdy, Farah Tamer Massoud, Seifeldin Ata Moheyeldin, Mahmoud Yehia Basha, Saleh Mohamed Hassan, Mohamed El-Kassas

**Affiliations:** 1 Internal Medicine Department, College of Medicine, Arab Academy for Science, Technology and Maritime Transport, Alexandria, Egypt; 2 College of Medicine, Arab Academy for Science, Technology and Maritime Transport, Alexandria, Egypt; 3 Endemic Medicine Department, Faculty of Medicine, Helwan University, Ain Helwan, Cairo, 11795 Egypt

**Keywords:** Virtual reality, Undergraduate medical education, Internal medicine, Simulation-based learning, Immersive learning, Virtual simulation

## Abstract

**Background:**

Virtual reality (VR) is increasingly recognized as a valuable tool in undergraduate medical education. It offers immersive, interactive environments that support experiential learning and skill development. However, its specific application in internal medicine education remains variably described. This scoping review aims to explore how VR is currently implemented in undergraduate internal medicine education, identify its educational outcomes, and highlight associated advantages, limitations, and gaps in literature.

**Methods:**

Following the Joanna Briggs Institute (JBI) methodology and PRISMA-ScR guidelines, we conducted a comprehensive search of eight databases, including PubMed, Scopus, Web of Science, and Embase. Studies were included if they addressed the use of VR in internal medicine education among undergraduate medical students. Data were extracted and analyzed thematically.

**Results:**

Of 1343 records screened, 9 studies published between 2017 and 2024 met the inclusion criteria. Studies originated from diverse countries and employed immersive and non-immersive VR technologies. Applications included teaching clinical reasoning, procedural skills, and emergency management. VR was associated with improved student engagement, satisfaction, and knowledge retention. Key advantages included scalability, safety, and cost-effectiveness. Limitations included technical challenges, limited physical interaction, and high initial costs. Few studies assessed long-term outcomes or included diverse, low-resource settings.

**Conclusion:**

VR is a promising adjunct to traditional internal medicine education, enhancing learner engagement and skill acquisition. However, its integration requires addressing technical barriers, ensuring faculty training, and expanding research to include diverse educational contexts and long-term effectiveness.

**Supplementary Information:**

The online version contains supplementary material available at 10.1186/s12909-025-08536-2.

## Background

Modern teaching approaches increasingly consider students’ unique learning styles, which strongly influence their understanding of medical sciences. Case-based learning (CBL), problem-based learning (PBL), simulation-based learning, and other innovative approaches engage students actively and connect theoretical knowledge with clinical scenarios [1]. These methods enhance competency, logical thinking, and clinical reasoning abilities. With the drive to provide meaningful clinical learning experiences, simulation has gained momentum as a method of delivering experiential, learner-centered environments [2]. 

The breadth of medical knowledge required of students continues to expand each year. Novel teaching methods are therefore needed to maximize study efficiency and bridge the gap between theoretical concepts and clinical practice. Early medical education often emphasizes basic science with limited exposure to patient-centered contexts, leading to fragmentation of knowledge, mismatch of competencies, and a restricted view of the patient. Innovative educational strategies are required to make medical training more engaging, scalable, and aligned with real-world demands [3]. 

Observational learning enhances procedural skills by providing immediate feedback, while simulation allows students to learn from mistakes in a safe setting, thereby reducing the risk of clinical errors [4]. Virtual reality (VR), a rapidly growing branch of simulation, is typically defined as a computer-generated three-dimensional environment that learners can interact with in real time. VR can be categorized into three levels of immersion: fully immersive, semi-immersive, and non-immersive systems [5]. Fully immersive VR employs head-mounted displays (HMDs), controllers, and motion detectors to place learners in realistic environments with a strong sense of “presence.” Semi-immersive VR utilizes large screens or projection-based systems that partially engage learners. In contrast, non-immersive VR relies on desktops or mobile devices, where interaction is limited but accessibility is greater [5]. The commercial release of affordable HMDs in 2014 greatly expanded the use of VR across medical education and healthcare training [6]. 

VR simulation provides exposure to realistic clinical scenarios and diverse patient outcomes, enabling students to develop diagnostic reasoning, receive real-time feedback, and practice repeatedly in a safe environment [7]. Across medicine and nursing, VR has been associated with increased learner confidence, improved performance, and individualized training opportunities [8, 9]. Virtual patient simulations are now being implemented in many medical schools worldwide, with adoption increasing rapidly in response to rising demand [10]. 

Internal medicine (IM) poses particular educational challenges. Clinical bedside teaching opportunities are usually constrained by many factors such as large student cohorts, variable inpatient exposure, and limited faculty resources. Yet IM is foundational, requiring mastery of complex clinical reasoning, multi-morbidity management, and care of undifferentiated presentations. VR can help address these challenges by recreating ward-based and emergency IM scenarios, offering repeatable and standardized learning opportunities while reducing reliance on scarce bedside teaching time [11]. 

Recent evidence highlights both the growth of VR in undergraduate medical education and the relative under-representation of internal medicine within this field. For example, a 2022 scoping review identified 114 VR studies in undergraduate medical education [12]. While a separate systematic review analysed 45 studies with 3,329 participants, only 10 of these studies (≈ 22%) involved internal medicine [13]. Similarly, a mapping of 126 randomized controlled trials (RCTs) in VR/AR education found that only one RCT focused on internal-medicine patient simulations [14]. On the other hand, promising IM-specific initiatives are emerging. A “virtual rounds” curriculum implemented during the COVID-19 pandemic with 29 clerkship students showed that 86% reported improved prerounding skills, 93% improved oral presentation skills, and 61% enhanced clinical reasoning [15]. Another large-scale IM course integrated immersive emergency VR scenarios for 529 senior medical students, demonstrating feasibility at scale and high learner engagement [16]. 

Taken together, these findings underscore both the significance and timeliness of focusing on internal medicine. While VR has been widely applied across medical education, IM remains underexplored despite its central role in shaping diagnostic reasoning and clinical decision-making skills. This scoping review, therefore, aims to synthesize current evidence on VR applications in undergraduate internal medicine education, outline reported educational outcomes, and highlight implementation strategies, advantages, limitations, and gaps in the literature.

## Methods

This scoping review was conducted in accordance with the Joanna Briggs Institute (JBI) methodology for scoping reviews [17] and reported following the PRISMA-ScR (Preferred Reporting Items for Systematic Reviews and Meta-Analyses Extension for Scoping Reviews) checklist [18]. The process comprised six stages: [1] identifying the research question [2], identifying relevant studies [3], study selection [4], charting the data [5], collating, summarizing, and reporting the results, and [6] stakeholder consultation.

### Stage 1: identifying the Research Question (RQ)

The primary objective of this review was to examine how VR is utilized in undergraduate internal medicine education. The following research questions guided the review:

1. How is VR used in undergraduate internal medicine education?
2. What are the main features of VR applications in this context?
3. What VR tools are available for undergraduate internal medicine education?
4. Which aspects of the internal medicine curriculum are targeted by VR?
5. What are the advantages and disadvantages of VR-based learning?

These research questions address VR in general. However, findings were analyzed and reported separately by immersion level (immersive, semi-immersive, and non-immersive). This approach acknowledges that immersion level may influence learner engagement, technical feasibility, and educational effectiveness.

### Stage 2: identifying relevant studies

A comprehensive search strategy was developed and applied across eight major electronic databases: PubMed, Scopus, Web of Science, Embase (Elsevier), SpringerLink, MEDLINE (Ovid), the Cochrane Central Register of Controlled Trials (Wiley), and the Education Resources Information Centre (ERIC). An initial limited search was performed in MEDLINE (Ovid) to refine the search terms. The keywords were classified into four sets covering core concepts of VR, internal medicine, undergraduate education, and clinical training (Appendix 1). Boolean operators and Medical Subject Headings (MeSH) were used to enhance sensitivity.

The final search string applied was:

- TS = (implement OR adopt OR disseminate* OR introduce*) AND
- TS = ("virtual reality" OR VR OR "virtual technology*" OR "virtual environment") AND
- TS = (health* OR "care" OR medical education* OR "Internal Medicine" OR ology OR clinic OR "General medicine") AND
- TS = (education OR training OR undergraduate*).

Additionally, backward reference searching of included studies was performed to identify potentially relevant articles not captured by database queries.

### Stage 3: study selection

The search results were uploaded into Rayyan and Covidence, two screening platforms used to manage citations and facilitate collaboration. Duplicates were removed, followed by a two-stage screening process:

Two independent reviewers screened titles and abstracts for eligibility, and two reviewers independently assessed full texts. Initial disagreements were resolved through discussion. In cases where consensus could not be achieved, the Internal Medicine consultants served as the adjudicator. All reviewers, including the mentor, were internal to the institution. No external reviewers were involved in study selection.

### Inclusion criteria

- Studies focusing on VR use in internal medicine education for undergraduate medical students.
- Full-text articles published in peer-reviewed journals.
- Studies reporting on immersive, semi-immersive, or non-immersive VR technologies.
- All primary research study designs (quantitative, qualitative, or mixed methods).
- Published between 2017 and December 2024.
- Studies published in English.

### Exclusion criteria

- Studies outside the domain of internal medicine.
- Non-primary studies (e.g., systematic reviews, meta-analyses, opinion papers, editorials).
- Conference abstracts and inaccessible full texts.

**Fig. 1:**
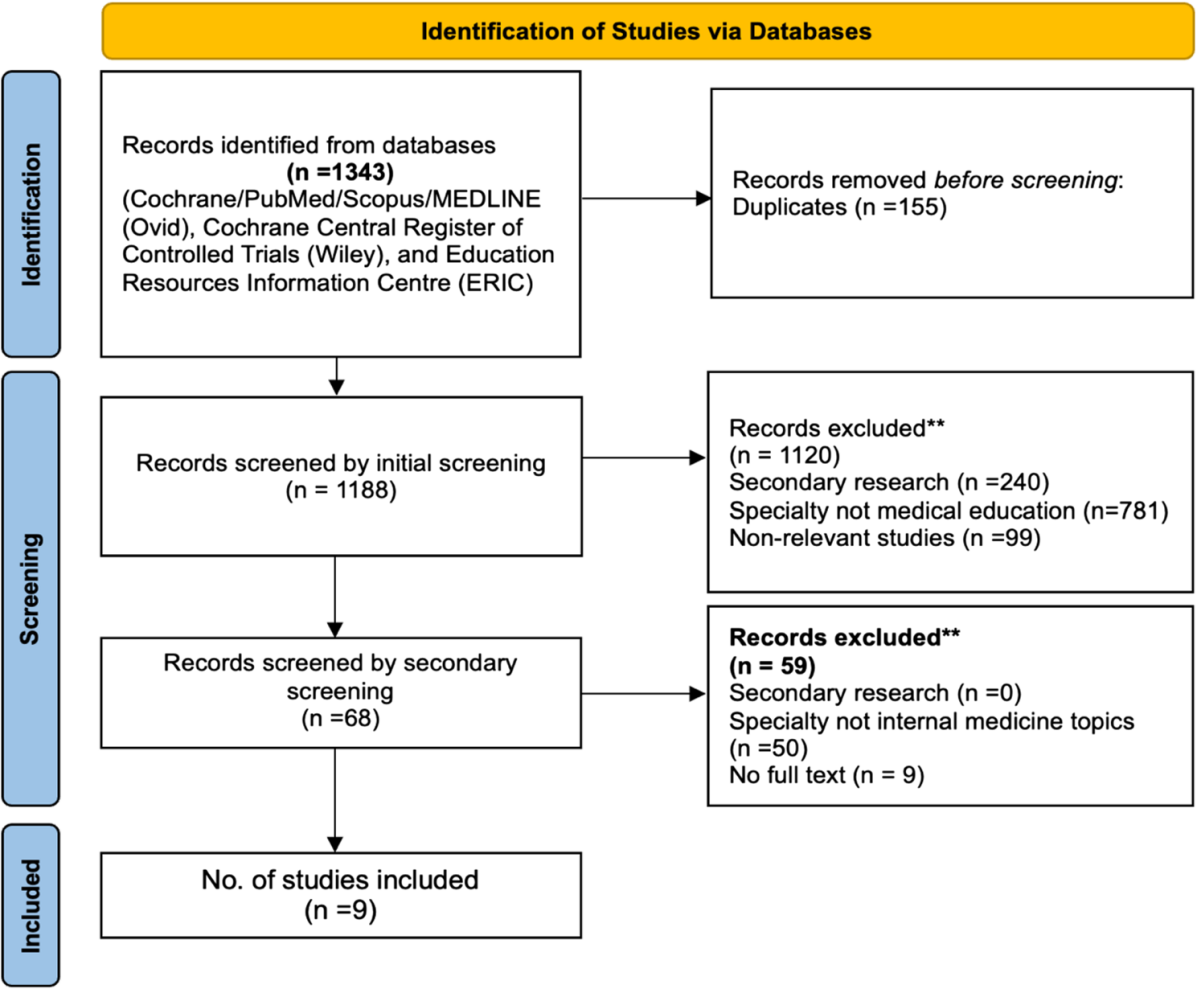
Preferred reporting items for systematic reviews and meta-analyses extension for scoping review (PRISMA-ScR) flow chart of study identification and selection. Source: Page MJ, et al. BMJ 2021;372:n71. doi: 10.1136/bmj.n71. This work is licensed under CC BY 4.0. To view a copy of this license, visit https://creativecommons.org/licenses/by/4.0/

The PRISMA-ScR flow diagram (Fig. 1) outlines the selection process.

### Stage 4: charting the data

#### Data extraction

A standardized data extraction form was developed and used to collect relevant details from each study. The data collected were summarized and reported using content analysis techniques according to the study’s purpose. The extracted data were categorized into bibliographic information, study characteristics, research questions, VR technology details, implementation characteristics, and outcomes. Further details of these data categories are illustrated in Table 1.

**Table 1:** Data extraction form

Category	Type of data
Bibliographic Information	
Author(s)	Name of the study authors
Year of publication	Year the study was published
Country of origin	Geographical location of the study
Objective of the study	Summary of the study’s purpose
Study characteristics	
Research questions or study goals	Specific aim or hypothesis of the study
Study design	Type of study (e.g., Randomized Controlled Trial (RCT), cohort)
Participants	Sample size and demographic details
Method of data collection	Techniques used (e.g., surveys, clinical assessments, task performance tests, interviews)
Research questions (RQ) and VR-specific characteristics	
Subjects (Demographics)	Description of medical student year level and background
Curriculum Integration (RQ1)	How VR was incorporated into the Internal medicine curriculum
Target population	Whether VR was used for preclinical or clinical students
VR Technology Characteristics	
VR description	Type of VR (Immersive vs. non-immersive)
VR goals	Intended Learning Outcomes (ILOs) of VR use
Target group	Specific Internal Medicine Subspeciality
Setting of use	Location of VR use (e.g., Simulation labs, bedside teaching)
Implementation characteristics	
Theoretical framework	Model used for VR Implementation
Integration approach	How VR was introduced into medical education
Technical and methodological requirements	
Hardware and software	Specifications of VR tools used (e.g., Meta Quest 3)
Number of VR sessions	Frequency and duration of VR training
Teaching methodologies	Learning techniques associated with VR use
Outcomes and key findings	
Educational outcomes	Knowledge retention, clinical reasoning, performance metrics
Advantages (RQ2)	Benefits of using VR in medical education
Disadvantages (RQ3)	Limitations or drawbacks of VR implementation
Challenges (RQ4)	Barriers to VR adoption (technical, financial, faculty-related)
Recommendations (RQ5)	Suggestions for improving VR-based education

### Stage 5: collating, summarizing, and reporting the results

A total of 1343 records were identified across eight databases. Following duplicate removal (*n* = 155), 1188 records underwent initial screening, resulting in the exclusion of 1120 studies due to irrelevance, non-primary research status, or non-medical educational contexts. Subsequently, 68 full-text articles were assessed for eligibility, leading to the final inclusion of nine studies published between 2017 and 2024.

Included studies represented diverse geographic locations, including Korea, Germany, Israel, the UK, the US, Saudi Arabia, and Chile. Various VR technologies were employed, classified broadly into immersive and non-immersive experiences. VR applications are predominantly focused on neurological examination training, clinical reasoning skills, procedural competencies, and emergency scenario management.

VR technologies were classified into three categories based on established definitions in past literature to ensure consistency across included studies:

- Immersive VR: technologies employing head-mounted displays (HMDs) or similar devices that fully occlude the user’s view of the physical environment, creating a sense of full engagement through 3D visualization and interaction [19, 20].
- Semi-immersive VR: systems that provide partial immersion through large-screen projections or 3D desktop environments, where users engage with the simulation but remain visually and physically aware of their real-world surroundings [21].
- Non-immersive VR: desktop- or web-based platforms where the user interacts with a virtual environment via a conventional computer screen, mouse, or keyboard, offering no perceptual immersion [19, 21].

When studies did not explicitly specify the immersion level, classification was inferred by examining the hardware setup (e.g., HMD, 360° headset, desktop computer), the mode of interaction (e.g., embodied vs. point-and-click navigation), and the descriptions of user experience (e.g., sense of presence, 360° perspective, or 2D interface) as recommended in prior research [20, 22].

Regarding educational outcomes, several studies (Han et al., Sultan et al.) reported significant improvements in skill acquisition and knowledge retention among VR users compared to traditional methods. However, other studies (Martinez et al.) noted modest improvements without statistically significant differences in objective structured clinical examination (OSCE) performance.

Student engagement and satisfaction were consistently higher with VR-based interventions. Walls et al. reported enhanced physiological engagement and subjective enjoyment, while Garber et al. indicated that the majority of students (95.1%) valued VR simulations and found them realistic.

Identified advantages of VR included immersive learning environments, cost-effectiveness, scalability, and the opportunity for safe, repetitive practice. Nevertheless, important limitations were also reported, such as technical difficulties (e.g., motion sickness and visual discomfort), limited physical interaction compared to traditional simulations, and substantial initial financial investment.

Notably, few studies assessed long-term outcomes or included diverse populations from resource-limited settings, highlighting critical gaps requiring further research.

### Stage 6: stakeholder consultation

To enhance the validity and applicability of the findings, a stakeholder consultation was conducted following the sixth JBI scoping review stage. Stakeholders included two experts in medical education and digital health, as well as faculty members with experience in internal medicine education.

The purpose of the consultation was to:

- Review the comprehensiveness of data synthesis and thematic categorization.
- Validate the practical implications of the findings for curriculum development.
- Identify overlooked areas or emerging priorities in VR-based education.
- Stakeholders provided qualitative feedback on the clarity and utility of the findings. They also contributed recommendations regarding future research priorities, particularly concerning the integration of VR in resource-limited settings and strategies for faculty development. Their input was incorporated into the final analysis and presentation of results.
- Ethical approval was not required as the review did not involve human subjects or primary data collection.

## Results

A total of nine studies were included in this review. Their characteristics are summarized in Appendices 2 & 3. Thematic analysis was conducted to identify key patterns in reported outcomes, which are presented under six themes: classification of VR immersion level, effectiveness in skill acquisition and knowledge retention, student engagement and satisfaction, advantages of VR, limitations of VR, and comparisons with traditional teaching methods.

### VR immersion level classification

Based on our immersion-level classification framework, six studies were categorized as immersive VR [23–28], two studies as non-immersive VR [29, 30], one as semi-immersive VR [31].

This classification enabled us to further explore whether the level of immersion influenced reported outcomes in engagement, knowledge retention, and skill acquisition across studies.

### Effectiveness of VR in skill acquisition and knowledge retention

Several studies demonstrated that VR significantly improved medical students’ clinical skills and knowledge retention. For example, Han et al. [23] found that students using a VR-based neurological examination teaching tool (VRNET) scored significantly higher in Neurologic Physical Exam (NPE) scores compared to those trained with standardized patients (SP) alone (*p* = 0.043). Similarly, Sultan et al. [25] reported that students exposed to 360° VR videos had higher mean scores in both knowledge retention and skills acquisition compared to those in the control group. Martinez et al. [31] also noted modest improvements in objective structured clinical examination (OSCE) performance among students using the Body Interact^®^ simulator, though the results were not statistically significant.

### Student engagement and satisfaction

VR was consistently reported as an engaging and enjoyable learning tool. Walls et al. [27] found that VR simulations elicited higher physiological engagement (e.g., increased heart rates) and subjective enjoyment compared to desktop-based simulations. Similarly, Macnamara et al. [24] highlighted that students found VR more immersive and enjoyable than high-fidelity manikins, though they preferred the latter for developing communication skills. Garber et al. [32] reported that 95.1% of students agreed or strongly agreed that VR simulations were valuable, and 92.5% felt the scenarios were like real-life clinical situations they would face.

### Advantages of VR

The studies identified several advantages of VR in medical education, including:

- Immersive learning environments: VR provided realistic, interactive scenarios that enhanced experiential learning [23, 25].
- Cost-effectiveness and scalability: VR was found to be a cost-effective alternative to traditional simulation methods, with lower setup costs and the ability to accommodate larger groups of students [26, 27].
- Safe and repetitive practice: VR allowed students to practice high-risk scenarios without consequences to real patients, enabling infinite repetition and feedback [23, 25].

### Limitations of VR

- Technical barriers: Issues such as motion sickness, difficulty focusing on high-detail screens, and the need for familiarity with VR technology and their learning curves were reported [24, 26, 33].
- Limited physical interaction: VR systems often could not replicate hands-on clinical skills, such as physical examinations and interpersonal communication [24, 30].
- High initial costs: While VR was cost-effective in the long term, the initial investment in equipment and software posed a financial barrier for some institutions [27, 31, 33].
- Immersive VR difficulties: Managing tools like head-mounted displays and spatial controllers can distract students from learning objectives (Rodriguez-Florido et al., 2024) [33].
- Additional challenges: The need for thoughtful, user-centered VR design is important for ensuring usability and minimizing cognitive overload (Rodriguez-Florido et al., 2024) [33].

### Comparisons of VR with traditional teaching methods

Several studies compared VR with traditional teaching methods, such as standardized patients (SP), high-fidelity manikins, and small-group discussions. Han et al. found that combining VR with SP training yielded better outcomes than SP alone, particularly in neurological examinations [23]. Middeke et al. reported that students using the EMERGE VR system scored significantly higher in clinical reasoning assessments compared to those in problem-based learning (PBL) groups [29]. However, Martinez et al. found no statistically significant difference in OSCE performance between students using VR and those participating in small-group discussions, suggesting that VR may not always outperform traditional methods [31]. 

### Gaps in literature

Despite the promising findings, several gaps were identified:

- Limited longitudinal data: Most studies did not assess long-term knowledge retention or skill transfer to real-world clinical settings [27, 31].
- Underrepresentation of diverse populations: Few studies included participants from low-income settings or explored the applicability of VR in resource-limited contexts [23, 25].
- Lack of standardized assessment tools: The validity and reliability of VR-based assessments were often questioned, with some studies relying on non-validated questionnaires or scoring systems [23, 30].

### Summary of findings

**Fig. 2:**
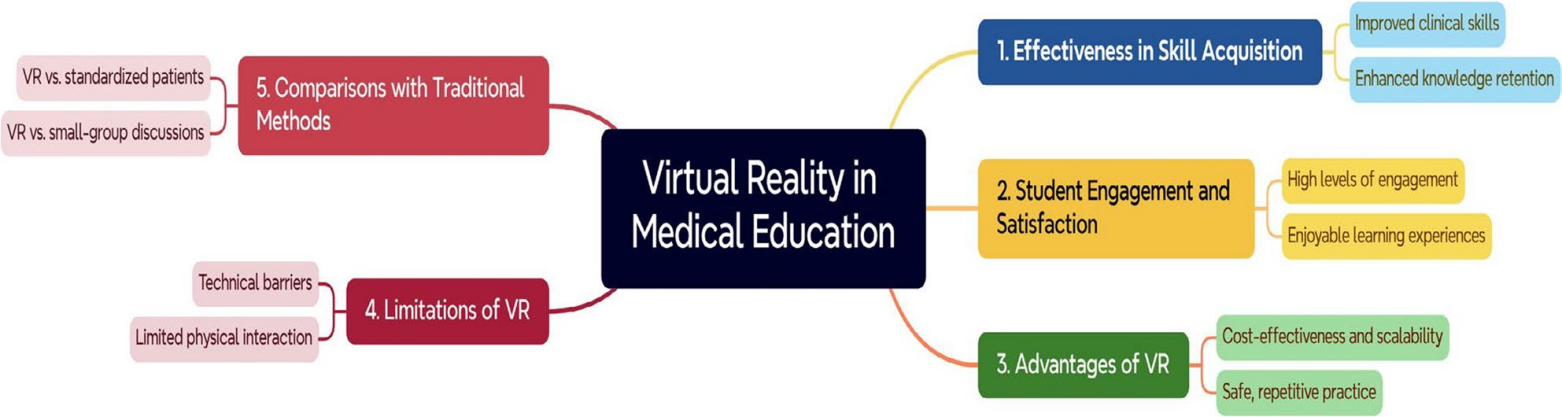
The key findings of the studies

The reviewed studies suggest that VR is a valuable tool for enhancing medical education, particularly in skill acquisition, engagement, and cost-effectiveness. However, its effectiveness varies depending on the context, and further research is needed to address technical limitations, improve assessment tools, and explore long-term outcomes. VR is best used as a complementary tool alongside traditional teaching methods rather than a standalone solution (Fig. 2).

## Discussion

The findings of this scoping review highlight the growing role of VR in internal medicine education, particularly in enhancing clinical skills, engagement, and cost-effectiveness. The reviewed studies demonstrate that VR can provide immersive, interactive learning environments that complement internal medicine’s traditional teaching methods. However, the effectiveness of VR varies depending on the context, and several challenges must be addressed to maximize its potential.

Our scoping review provides insight into the existing evidence as well as gaps in the use of VR in internal medicine medical education and provides recommendations for future research in this area.

The effectiveness of VR in improving clinical skills and knowledge retention is supported by multiple studies. For instance, Han et al. [23]and Sultan et al. [25] reported significant improvements in skill acquisition among students using VR compared to traditional methods. These findings align with previous research by Johnson et al., emphasizing the value of immersive technologies in medical training [34]. However, Martinez et al. (2023) found no statistically significant difference in OSCE performance between VR and small-group discussions, suggesting that VR may not always outperform traditional methods. This inconsistency underscores the need for further research to identify the specific context in which VR is most effective.

VR has been widely adopted in surgical training, where it has been found to reduce injuries, speed up procedures, and enhance overall outcomes. It has since been incorporated into other surgical procedures with outstanding success [35].

VR can be used to train clinicians in complex procedures such as transvenous lead extraction, is effective in cardiopulmonary resuscitation training, and can improve communication skills, critical thinking, and clinical decision-making [21, 22, 36]. 

VR has also outperformed realistic simulations. One study randomly assigned 84 nursing students to either a virtual or actual nursing simulation. Learning transfer was consistent, with no significant difference in performance between groups. However, the simulation in the VR group was determined to be significantly cheaper [37]. 

Our findings concur with review studies that have already been published and demonstrate the benefits of VR [38–40]. Jiang et al. surveyed the various uses of VR in medical education. Similar to our results, VR was more effective in managing clinical scenarios and in clinical knowledge [41]. 

### Engagement and satisfaction

The high levels of student engagement and satisfaction reported in studies such as Walls et al. [42] and Garber et al. [28] reflect the potential of VR to create enjoyable and motivating learning experiences. These findings are consistent with the broader literature on technology-enhanced learning, which highlights the importance of engagement in improving educational outcomes by Brown et al. [4]. However, the novelty effect of VR may influence student perceptions, as noted by Walls et al. [42] and longitudinal studies are needed to determine whether these positive attitudes persist over time.

### Advantages and limitations

The studies identified several advantages of VR in medical education, including:

- Immersive learning environments: VR provided realistic, interactive scenarios that enhanced experiential learning [23, 25].
- Cost-effectiveness and scalability: VR was found to be a cost-effective alternative to traditional simulation methods, with lower setup costs and the ability to accommodate larger groups of students [26, 27].
- Safe and repetitive practice: VR allowed students to practice high-risk scenarios without consequences to real patients, enabling infinite repetition and feedback [23, 25].

VR simulation education was found to be 22% more efficient than high-fidelity simulation education. The cost of the VR was determined to be 40% lower than that of the high-fidelity simulation [43, 44]. 

### Comparisons of VR with traditional teaching methods

Several studies have compared VR with traditional teaching methods, including standardized patients (SP), high-fidelity manikins, and small-group discussions. Han et al. found that combining VR with SP training yielded better outcomes than SP alone, particularly in neurological examinations [23]. Middeke et al. reported that students using the EMERGE VR system scored significantly higher in clinical reasoning assessments compared to those in problem-based learning (PBL) groups [29]. However, Martinez et al. found no statistically significant difference in OSCE performance between students using VR and those participating in small-group discussions, suggesting that VR may not always outperform traditional methods [31]. 

### Implications for practice

The findings show that VR is best used as a supplement to traditional educational methods rather than as a complete solution. Educators should consider adding VR into select areas of medical instruction, such as high-risk scenarios or procedural skills, where its immersive characteristics can be most beneficial. In addition, institutions should invest in faculty training and technical support to solve the obstacles of VR deployment.

### Global VR incorporation into the curriculum of medical schools

VR simulation is used in medical schools as well as in postgraduate education around the world. Practical implementation and curriculum integration vary depending on many factors. The VR platform availability, cost per student, and institutional readiness.

VR was applied in the educational practice involving undergraduate medical students across 25 countries. However, most countries involved (97%) were from Europe, North America, and Asia. This regional bias might be due to the uneven distribution of digital medical education resources across the world, which would influence the local medical students’ access to education in underdeveloped areas [10]. 

Stanford University has developed VR simulations for medical students that allow them to practice clinical skills and patient interactions in a controlled environment. Harvard Medical School leverages VR to create realistic patient scenarios for its students, helping them to practice and refine their clinical skills. The University of Michigan uses VR to enhance the teaching of clinical skills, particularly those relevant to internal medicine, allowing students to experience real-life scenarios. Many medical schools use VR for various applications, including surgical training, procedural simulations, and even mental health assessments. Drexel University New York University (NYU) University of California, San Francisco (UCSF) Western University (Canada), and the University of Sydney. As technology evolves, more institutions are likely to adopt these tools, making VR an integral part of medical education.

The University of Northampton and Oxford University Hospitals. As well as hospitals and universities, VR systems are also being used across healthcare systems, with Health Education England, East of England supporting the delivery of VR simulation across 18 NHS trusts from August 2019. Evidence for the educational effectiveness of VR in medical students’ knowledge or skills was sufficient as per Kirkpatrick’s model of outcome evaluation.

However, the highly shareable feature of digital resources has been considered to provide an opportunity to address the need for a fair learning system for medical students and promote equity in medical education globally [45, 46]. 

Even in resource-limited settings, the application of VS educational systems/platforms could help to promote medical learning by reducing instructor costs and laboratory materials. Along with the advancement and expansion of computer technology, VS has been believed as a less expensive and more accessible alternative for undergraduate medical education, allowing for its wide application in low- and middle-income countries [47, 48]. 

Qingming et al. showed the distribution of studies reporting the use of VR in undergraduate medical education on different continents. This study revealed that Africa is the least represented continent without any real contribution to VR research [40]. 

As such, VR simulation can fit around institutional needs as required. Though the specific examples mentioned here refer only to education and training, VR simulations are also being used in other areas. The standardized and objective nature of scenarios has allowed various institutions to implement assessments and recruitment programs using VR [49]. In recruitment, VR scenarios are being used as a proxy for clinical competency and form a basis for ongoing interviews. This facilitates recruitment locally as well as overseas, as technology works in any setting and does not need expert faculty to run.

### Study limitations

This scoping review has some limitations that should be acknowledged. First, the small number of studies included (*n* = 9) limits the robustness of the conclusions drawn. Second, there was considerable geographical concentration, with most studies originating from high-income or well-resourced settings. This limits the generalizability of our findings to lower-income countries or regions with fewer resources. Third, the substantial methodological variability among included studies, such as differences in study designs, outcome measures, and assessment tools, poses challenges for comprehensive synthesis and comparison. Additionally, most of the studies evaluated only immediate or short-term educational outcomes, highlighting a gap in evidence regarding the long-term effectiveness of VR interventions. Finally, the review was limited to studies published in English, potentially omitting relevant research published in other languages. These limitations should be considered when interpreting our findings and emphasizing the need for future research addressing these gaps.

### Future research directions

Future research in VR for undergraduate internal medicine education should address several critical areas to enhance its effectiveness and generalizability. First, longitudinal studies are needed to evaluate the long-term impact of VR on knowledge retention and clinical performance, as most existing research focuses only on short-term outcomes. Second, there is a pressing need to include more diverse populations, particularly participants from low-income or resource-limited settings, to assess the global applicability and equity of VR-based education. Third, the development and use of standardized, validated assessment tools are essential, as many current studies rely on unverified scoring systems, raising concerns about the reliability of their findings. Fourth, comparative research should be undertaken to evaluate the effectiveness of VR relative to traditional teaching methods across various educational contexts and medical specialties. Finally, technical barriers such as motion sickness, restricted physical interaction, and insufficient faculty training should be systematically investigated and mitigated to facilitate broader adoption and integration of VR technologies in medical curricula.

## Conclusion

This scoping review highlights the potential benefits and limitations of integrating VR into undergraduate internal medicine education. The reviewed studies suggest that VR effectively enhances learner engagement, satisfaction, and clinical skill acquisition through immersive, interactive, and safe practice environments. Despite these promising outcomes, its effectiveness remains context-dependent, influenced by technical challenges, the need for substantial initial investments, and limitations in replicating hands-on physical interactions.

To maximize the educational value of VR, future research should focus on evaluating long-term educational impacts, developing standardized and validated assessment tools, and including more diverse populations, particularly from low-resource settings. Currently, VR serves best as a complementary educational tool rather than a replacement for traditional teaching methods. Continued technological advancements, coupled with thoughtful curricular integration and faculty training, will be crucial to overcoming existing barriers and fully realizing VR’s potential to transform medical education globally.

## Supplementary Information

Supplementary Material 1.

## Data Availability

The data that support the findings of this study are available from the corresponding author upon reasonable request.

## Abbreviations

VR: Virtual Reality
IM: Internal Medicine
CBL: Case-Based Learning
PBL: Problem-Based Learning
HMD: Head-Mounted Display
PRISMA-ScR: Preferred Reporting Items for Systematic Reviews and Meta-Analyses extension for Scoping Reviews
JBI: Joanna Briggs Institute
RCT: Randomized Controlled Trial
OSCE: Objective Structured Clinical Examination
SP: Standardized Patient
ILOs: Intended Learning Outcomes
