# Supplementary material for: Incorporating virtual reality in undergraduate internal medicine education: a scoping review of current evidence and implementation strategies
Source: BMC Med Educ. 2026 Feb 10;26:334. doi: 10.1186/s12909-025-08536-2 (PMC12930858; doi:10.1186/s12909-025-08536-2)
Supplement: Supplementary file 1 — Supplementary Material 1. [file 12909_2025_8536_MOESM1_ESM.docx]

**Appendix 1**

Search Strategy and Keyword Classification

| Set | Key concepts | Related terms |
| --- | --- | --- |
| Set 1 | Virtual reality (VR) | VR, Virtual Reality,  virtual environment |
| Set 2 | Internal medicine | Internal medicine, General medicine, Clinical, -ology |
| Set 3 | Undergraduate | Education, undergraduate |
| Set 4 | Medical students | Clinical training, MBBS, clerkship |

**Appendix 2**

Overview of the nine references that met our inclusion criteria

|  | Authors, Year of publication and country | **Results** | **Limitations** |
| --- | --- | --- | --- |
| 1 | Han SG et. al., 2021, Republic of Korea. | - **Student Satisfaction & Realness**: No statistical differences between SP and SP with VRNET groups. - **Neurologic Physical Exam (NPE) Performance**: SP with VRNET group scored significantly higher (3.81 ± 0.92) than SP group (3.40 ± 1.01, p = 0.043). | - **Assessment Limitation**: Unable to measure students' neurological examination skills within each group. - **Unclear Practical Significance**: While NPE score differences were statistically significant, their real-world impact is uncertain. - **Validity & Reliability Concerns**: The questionnaire and NPE score’s validity and reliability were not confirmed. - **Recognition of Abnormal Findings**: The study did not assess whether students correctly identified abnormal neurological findings. - **Generalizability Issue**: Results may not directly apply to real clinical settings due to differences from controlled CPX environments. |
| 2 | Angélina Middeke et. al.  ,2018,Germany | - **Key Feature Examination**: EMERGE group (62.5%, IQR: 17.7) scored significantly higher than the PBL group (54.2%, IQR: 21.9, p = 0.015). - **Training Phase Performance**: No significant difference between groups in cases discussed in both instructional formats. - **Final EMERGE Session**: EMERGE group outperformed PBL group in total score for all four cases and in three of four cases for final diagnosis and correct therapeutic interventions. | - **Limited Generalizability**: Monocentric study design and focus on fifth-year students restrict applicability to other student groups and medical fields. - **Lack of Randomization**: No participant randomization, potentially affecting study reliability. - **Confounding Factor**: Learning time differences may have influenced results. - **Unassessed Digital Feedback Use**: Unable to determine how much EMERGE group students utilized digital feedback. - **Selection Bias**: Choice of key feature cases may have unintentionally favored the EMERGE group. |
| 3 | Arie Oliven et al., 2021, Israel. | - **Examination Tool:** VP system is valid and reliable for assessments. - **Independent Training**: Supports student learning during ward-based training and outside formal settings. - **Flexible Learning**: Useful when not in classes, wards, or clinics, especially during social distancing. | - **Not reported.** |
| 4 | Risheka Walls et al., 2024, UK. | - **Enhanced Engagement**: VR simulations are more engaging and enjoyable than traditional desktop simulations. - **Physiological Engagement**: Students showed higher heart rates and focused attention during VR sessions. - **Skill Development**: VR was effective for training in emergency scenario management. - **Scalability & Cost-Effectiveness**: Enables large-group training in a safe environment. - **Realistic Critical Scenario Training**: Provides immersive exposure to life-threatening emergencies not easily accessible in clinical settings. - **Educational Value**: VR is a valuable complement to traditional medical training, especially for emergency situations. | - **Technical Limitations**: VR struggles to fully replicate in-person simulations, limiting its effectiveness in developing hands-on technical skills. - **Curriculum Integration Challenges**: Incorporating VR into existing medical curricula requires significant planning and adaptation. - **Familiarity & Training Barriers**: Both students and educators need time to adapt to VR technology, potentially slowing adoption |
| 5 | Alexandra Frances Macnamara et al., 2021, UK. | - **SimMan 3G High-Fidelity Manikin**: Preferred by 18 out of 19 participants for better clinical preparation, increased confidence, and a more impactful learning experience. - **OMS Virtual Reality**: Praised for immersion, engagement, and beneficial guidance, particularly for early-year students developing clinical skills. - **Complementary Use**: The Study highlights the potential of integrating both technologies in medical education. - **Recommendation**: Further research needed to optimize their integration into curricula. | - **Communication Skills Gap**: VR lacks opportunities for practicing verbal communication and handover skills essential in clinical settings. - **Realism Limitations**: Some students felt VR scenarios did not fully replicate real clinical situations, particularly regarding time pressure and patient interactions. - **Limited Physical Interaction**: Inability to perform physical examinations in VR restricts hands-on clinical skill development. |
| 6 | Lama Sultan et. al., 2019, Saudi Arabia. | - **Experiential Learning**: Supports learning by doing through rich, interactive, and engaging contexts. - **Increased Motivation**: Enhances student interest and engagement. - **Improved Retention & Skills**: Effectively supports knowledge retention and skill acquisition. | - **Gender Imbalance**: Fewer female participants, with some dropping out due to logistical issues. - **Limited Student Diversity**: Involving only students from the same academic level may have restricted broader perspectives; inclusion of different levels could provide better insights. |
| 7 | Patrick W. Chang et. al., 2017, US. | - **Clinical Preparation**: 79% of students found VR helpful and showed interest in future use if technological barriers were addressed. - **Learning Outcomes**: No significant difference between VR and control groups on an 8-item quiz, confirming VR did not hinder learning. - **Cost-Effective Integration**: VR video was affordably incorporated into the standardized patient program. - **Feasibility & Benefits**: VR is a viable adjunct to traditional education, enhancing early patient exposure without compromising outcomes. | - **Limited Sample Size**: Small participant group (N = 183) restricts generalizability and broader conclusions on VR effectiveness. - **Technological Barriers**: challenges in overcoming technical issues and ensuring proper training for both students and instructors. |
| 8 | Felipe T. Martinez et. al., 2023, Chile. | - **Effectiveness**: A clinical virtual simulator (Body Interact®) showed a modest but non-significant improvement in OSCE performance. - **Comparison with Small-Group Discussions**: No significant advantage over small-group discussions in academic performance or adherence to guidelines. - **Intervention Duration**: Short intervention limited knowledge retention and skill acquisition. - **Future Recommendations**: Longer evaluation periods and diverse clinical scenarios needed to better assess effectiveness. | - **Short Intervention Period**: Limited training duration may have restricted knowledge retention and skill development. - **Hawthorne Effect**: Participants' awareness of being observed may have influenced their performance, potentially affecting study results. |
| 9 | Adam M. Garber et. al.., 2024, US. | - **Effectiveness**: VR is an effective, acceptable, and cost-efficient tool for teaching urgent clinical situation management, specifically Core Entrustable Professional Activity 10 skills. - **Student Satisfaction**: Over 90% of students agreed VR prepared them for intern responsibilities. - **Educational Value**: Provides a realistic and flexible learning environment while being less resource-intensive than traditional high-fidelity simulations. - **Future Integration**: Findings support further incorporation of VR in medical training to enhance student preparedness and learning experiences. | - **Faculty Time Requirement**: Despite being less time-intensive than high-fidelity simulations, VR still requires faculty involvement for development and oversight. - **Learning Curve**: Students may need time to adapt to VR technology, potentially slowing the initial training process. |

**Appendix 3:**

Detailed characteristics of the nine references that met our inclusion criteria

<https://docs.google.com/spreadsheets/d/185JRxIaXO_UP5XEkyYTXcST10MWXTFp0/edit?usp=sharing&ouid=110075354509857394503&rtpof=true&sd=true>
